# Measuring the Interprofessional Health of the Pediatric Cardiovascular Operating Room Work Environment

**DOI:** 10.1097/pq9.0000000000000737

**Published:** 2024-06-11

**Authors:** Jason M. Thornton, Jean A. Connor, Patricia A. Dwyer, Courtney L. Porter, Lauren P. Hartwell, Zachary DiPasquale, Araz Chiloyan, Patricia A. Hickey

**Affiliations:** * From the Nursing/Patient Services, Cardiac Intensive Care Unit & Cardiovascular Operating Rooms, Cardiovascular and Critical Care, Patient Services, Boston Children’s Hospital, Boston, Mass.; † Nursing Research, Cardiovascular, Critical Care and Perioperative, Patient Services, Boston Children’s Hospital, Harvard Medical School, Boston, Mass.; ‡ Nursing Research, Perioperative and Satellite, Patient Services, Department of Nursing, Boston Children’s Hospital, Boston, Mass.; § Cardiovascular, Critical Care and Perioperative, Patient Services, Boston Children’s Hospital, Boston, Mass.; ¶ Department of Cardiology, Boston Children’s Hospital, Boston, Mass.; ‖ Nursing and Patient Care Operations, Boston Children’s Hospital, Harvard Medical School, Boston, Mass.

## Abstract

**Introduction:**

Pediatric cardiac surgery is complex and has significant risk, requiring interprofessional teamwork for optimal outcomes. Unhealthy work environments have been linked to poor patient outcomes, staff dissatisfaction, and intention to leave. We describe the interprofessional health of pediatric cardiovascular operating room (CVOR) work environments in the United States and the establishment of a healthy work environment (HWE) benchmark score.

**Methods:**

Utilizing the American Association of Critical Care Nurses Healthy Work Environments Assessment Tool (HWEAT), interprofessional staff from 11 pediatric CVORs were surveyed. Responses were aggregated, summarized, and stratified by role to examine differences. The following phase used an e-Delphi approach to obtain expert consensus on a benchmark target.

**Results:**

Across 11 centers, 179 (60%) completed surveys were reviewed. The interprofessional mean HWEAT score was 3.55 (2.65–4.34). Mean scores for each standard were within the “good” range. Participants reported the highest scores for effective decision-making, with a mean of 3.69 (3.00–4.20). Meaningful recognition scored lowest, mean 3.26 (2.33–4.07). When stratified, surgeons reported higher overall HWE scores (M = 3.79, SD = 0.13) than nurses (M = 3.41, SD = 0.19; *P* = 0.02, two-tailed). The proposed benchmark was 3.50.

**Conclusions:**

This is the first time the American Association of Critical Care Nurses HWEAT has been used to describe the interprofessional health of work environments in pediatric CVORs in the United States. The targeted benchmark can support pediatric CVOR improvement strategies. Creating and sustaining an HWE is an interprofessional opportunity to support high-quality patient outcomes and clinical excellence.

## INTRODUCTION

Pediatric cardiac surgery is complex and associated with significant risk, relying on strong interdisciplinary teamwork for optimal outcomes and avoiding patient harm. As in most pediatric cardiovascular surgery programs across the United States, the cardiovascular operating room (CVOR) interprofessional team comprises clinicians highly specialized in pediatric cardiac surgery.^1,2^ For over a decade, the American Association of Critical Care Nurses (AACN) has championed a Healthy Work Environment (HWE) to support optimal patient outcomes and clinical excellence.^3^ The AACN HWE framework, with its six evidence-based standards, interacts dynamically to promote clinical and operational excellence for optimal patient outcomes (**Supplemental Digital Content 1**, which describes AACN standard for establishing and sustaining healthy work environments, http://links.lww.com/PQ9/A562). In 2009, guided by this framework, the AACN released the HWE Assessment Tool (HWEAT), developed to assist hospitals in determining pathways to establish and sustain HWEs.^4^ The HWEAT has undergone reliability and validity testing in various clinical settings with a reported Cronbach α value of 0.96.^4–7^ Connor et al^5^ demonstrated the interprofessional validity of this tool in measuring the health of the work environment across all disciplines in the healthcare setting. However, there continues to be limited information evaluating specialty area work environments despite several studies finding the HWEAT valid and reliable.^5,7^

In operating rooms (ORs), specifically CVORs, across the United States, workforce stability is of concern. On average, nurses in the perioperative specialty are older than nurses in other specialties.^8^ Messina et al^9^ noted that, on average, perioperative nurses are 5 years older and more likely to retire sooner than those in other specialties, adding additional concern for future perioperative staffing needs. Messina et al^9^ also noted that there had never been a large influx of nurses into the perioperative specialty, and an impending shortage has been noted since the early 1990s. They predict that approximately 20% of perioperative nurses will retire five years after publication; by September 2016, Ball et al^10^ noted that 65% of perioperative nursing leaders would retire within 10 years or less. Many hospitals and healthcare systems already struggle to adequately staff their ORs because of this perioperative specialty nursing shortage.^11^ Although no published data are specific to pediatric CVORs, a nationwide review of current job postings highlights hospitals’ challenges in hiring and retaining OR nurses within this highly specialized setting.

An inadequate staffing model has been linked with a poor work environment, which includes poor interprofessional collaboration, ineffective communication and leadership, and a lack of mutual respect and recognition.^10,12^ Establishing and sustaining HWEs is essential for promoting quality and safety in healthcare.^13^ A growing body of literature suggests that HWEs positively impact healthcare organizations and patient outcomes.^14–16^ HWEs are associated with decreased patient mortality and nurse intent to leave.^13,14,17^ Olds et al^14^ found that one SD increase in positive perception of the nurse work environment was associated with an 8.1% decrease in odds of mortality (odds ratio 0.919, *P* < 0.001). Factors within work environments, such as improved staffing and participation in decision-making, were associated with decreased nurse turnover rates.^17^

The HWEAT is considered a generalizable survey for any hospital or unit to identify areas for improvement.^6^ AACN outlines mean scores by providing cut points to determine if and where respective work environments need improvement. These cut points become the basis for benchmarking, an emerging healthcare management concept. Benchmarking in healthcare is defined as a collaborative process of measuring and comparing results against other performers to evaluate organizational performance.^18^ The benchmark reflects best practices and drives quality improvement (QI) initiatives.^19^ Since 2010, AACN has maintained an aggregate of HWEAT scores, including overall mean scores, which can serve as a benchmark. However, these data are not stratified by specialty, so this benchmark does not capture unique influences in the pediatric CVOR.

Despite evidence supporting the relationship between HWEs and positive organizational outcomes and the documented challenges of inadequate staffing models while working in the OR, little is known about the health of pediatric CVORs. There is no AACN HWEAT benchmark target for pediatric CVOR work environments in the United States. Identifying a benchmark will support the development of targeted interventions to improve this unique work environment and foster nurse retention.

### Specific Aims

The primary aim of this study is to describe and assess the health of the work environments in pediatric CVORs in the United States. The secondary aim is to identify a target benchmark to guide improvement strategies.

## METHODS

### Clinical Setting

Inclusion criteria were any pediatric CVORs in freestanding children’s hospitals in the United States. with annual cardiac surgical volume greater than 50 cases that participate in the Consortium for Congenital Cardiac Care—Measurement of Nursing Practice (C4-MNP). C4-MNP is an international collaboration of 45 cardiovascular programs in pediatric hospitals across the United States, Canada, and the Middle East. The collaboration aims to identify nursing care actions for measurement in the highly complex pediatric cardiovascular patient environment.^20,21^ The target population for the study included all interdisciplinary CVOR team members participating in the consortium and included registered nurses, surgical scrub technologists, clinical assistants, physician assistants, attending cardiac surgeons, attending cardiac anesthesiologists, and perfusionists.

### Design and Procedures

This QI project utilized an electronic survey design to assess the health of pediatric CVORs in the United States. A second phase of the project used an e-Delphi approach to obtain consensus from a group of experts on a pediatric CVOR HWE benchmark target. The co-chairs of the Nursing Scientific Review subcommittee of the Nursing Research Council determined that this project met the criteria for a QI activity. Per policy, projects that meet the criteria for QI do not require additional IRB review or approval as exempt. Following approval, the Principal Investigator proposed the project to members of C4-MNP, with 11 of the 32 US-based sites expressing interest in participating and identifying a specific CVOR nursing representative. Ten of 11 sites accepted the lead site’s determination of QI activity, with the remaining site receiving this designation from their IRB. A conference call for representatives included a project review, further background on the HWEAT, clarification of sites’ expectations, and the opportunity to ask questions. A follow-up email summarizing the call included links providing background information to prepare representatives for discussion with their teams.

The free, publicly available, and validated AACN HWEAT was used to assess the health of pediatric CVORs. Written permission was received from AACN to take the survey offline. A REDCap database was built using the AACN HWEAT (**Supplemental Digital Content 2**, which describes AACN Healthy Work Environment Assessment, http://links.lww.com/PQ9/A563), with the text of the 18 statements replicated exactly as they appeared in the AACN online version (www.aacn.org/hwe). Response choices for each of the 18 statements were also replicated.

Each confirmed site received an electronic form letter encouraging participation and introducing the survey to their staff. The site representative disseminated the form letter and link to their interdisciplinary CVOR team to participate in the assessment. To ensure confidentiality, each survey participant received a survey link via email. The link included two data collection instruments: a demographic questionnaire and the HWEAT.

Participants identified their clinical role and then proceeded to three demographic questions: years of pediatric CVOR experience, highest nursing degree (if applicable), and RN specialty certification. The six evidence-based standards assessed in the HWEAT are skilled communication, true collaboration, effective decision-making, appropriate staffing, meaningful recognition, and authentic leadership.^3^ Individuals respond to statements using a five-point Likert scale ranging from 1 (strongly disagree) to 5 (strongly agree). Mean scores from 1.00 to 2.99 suggest “needs improvement”; 3.00 to 3.99 suggests “good”; and 4.00 to 5.00 suggests “excellent.” Scores are calculated for each standard and the overall response.^4^

All responses were returned electronically, saved by a confidential site number in a secure, password-protected database, and de-identified. Each site representative received weekly reminder emails with their team’s response rate. The survey opened in December 2017 and closed for all sites after 1 month.

During the project’s second phase, the e-Delphi methodology was used to identify a target benchmark to guide improvement strategies in pediatric CVORs. The Delphi technique is a widely accepted systematic approach for formal consensus development.^22^ When this technique utilizes electronic survey technology, it is called e-Delphi. The e-Delphi approach allowed the project lead to obtain a collective view from a geographically diverse group of CVOR stakeholders.^23^ The e-Delphi approach allowed participants to propose benchmark targets without being influenced by others. Two short surveys elicited input regarding an HWE benchmark target from the 11 participating sites. In the first e-Delphi round, the survey included a summary of the HWEAT aggregate mean and range of scores collected, and participants were asked to propose a CVOR HWE benchmark. During the second e-Delphi round, the survey included the round one de-identified answers and asked one question: “Please propose a CVOR HWE benchmark after considering the round one answers of other pediatric CVOR experts.”

Each site received an individualized report comparing its results to the aggregate. Once a benchmark target was agreed upon, the participating sites were reconvened. Strategies to improve scores in each of the six essential standards were presented. The group generated additional ideas, and the feasibility of implementation was reviewed. One-on-one discussions were offered for interested sites, particularly those who scored lower than the benchmark, to review the findings, identify the most impactful interventions, and convene a focus group to dive deeper and identify themes.

Participant responses from the HWEAT were analyzed for individual sites and aggregated for the overall AACN HWEAT total score and by standard. Data were stratified by nursing and physician discipline. A two-sample *t* test was used to compare the mean scores of surgeons to nurses (*P* ≤ 0.05). Following the first e-Delphi round, the proposed benchmark scores were summarized by ranking all scores from lowest to highest. After the second e-Delphi round, the score that gained more than 90% agreement was reported as the target benchmark for pediatric CVOR.

## RESULTS

The total number of clinicians responding to the survey was 179, with an overall response rate of 60%. Of the 179 clinicians who took the survey, 36% (65) were RNs, 16% (28) anesthesiologists, 15% (27) perfusionists, 11% (19) surgical scrub technologists, 11% (19) surgeons, 4% (7) physician assistants, 5% (9) responded other, and the remaining 2% (3) did not note their clinical role (Table 1).

**Table 1.** Demographic Characteristics of Participants (n = 179)

Characteristic	N (%)
Role	
Anesthesiologist	28 (15.6)
Perfusionist	27 (15.1)
Physician assistant	7 (3.9)
Registered respiratory therapist	2 (1.1)
Registered nurse	65 (36.3)
Surgeon	19 (10.6)
Surgical scrub technologist	19 (10.6)
Other (certified registered nurse anesthetist, RN coordinator or manager, nurse practitioner, surgical assistant)	9 (5.1)
Did not respond	3 (1.7)
Years of pediatric CVOR experience	
0–2	58 (32.4)
3–5	33 (18.4)
6–10	31 (17.3)
11–15	15 (8.4)
More than 15	36 (20.1)
Did not respond	7 (3.9)
Highest nursing degree (n = 65)	
Diploma	3 (4.6)
Associate’s degree	9 (13.8)
Bachelor’s degree	47 (72.3)
Master’s degree	3 (4.6)
Degree in a field other than nursing	1 (1.5)
Did not respond	2 (3.1)
Nursing specialty certification (n = 65)	
Yes	36 (55.4)
No	27 (41.5)
Did not respond	2 (3.1)

Of the respondents, 32% (58) had 0–2 years of experience, 18% (33) had 3–5 years of experience, 17% (31) had 6–10 years of experience, 8% (15) had 11–15 years of experience, 20% (36) had more than 15 years of experience, and 4% (7) did not note their years of experience. Of the respondents who self-identified as a registered nurse, 72% (47) were BSN prepared, 5% (3) had a Master’s degree, 14% (9) had an Associate’s degree, 5% (3) were Diploma graduates, 2% (1) had a degree in a field other than nursing, and 3% (2) did not note what level of nursing education they had achieved. Over half (55%) indicated they had an RN specialty certification (Table 1).

**Fig. 1.**
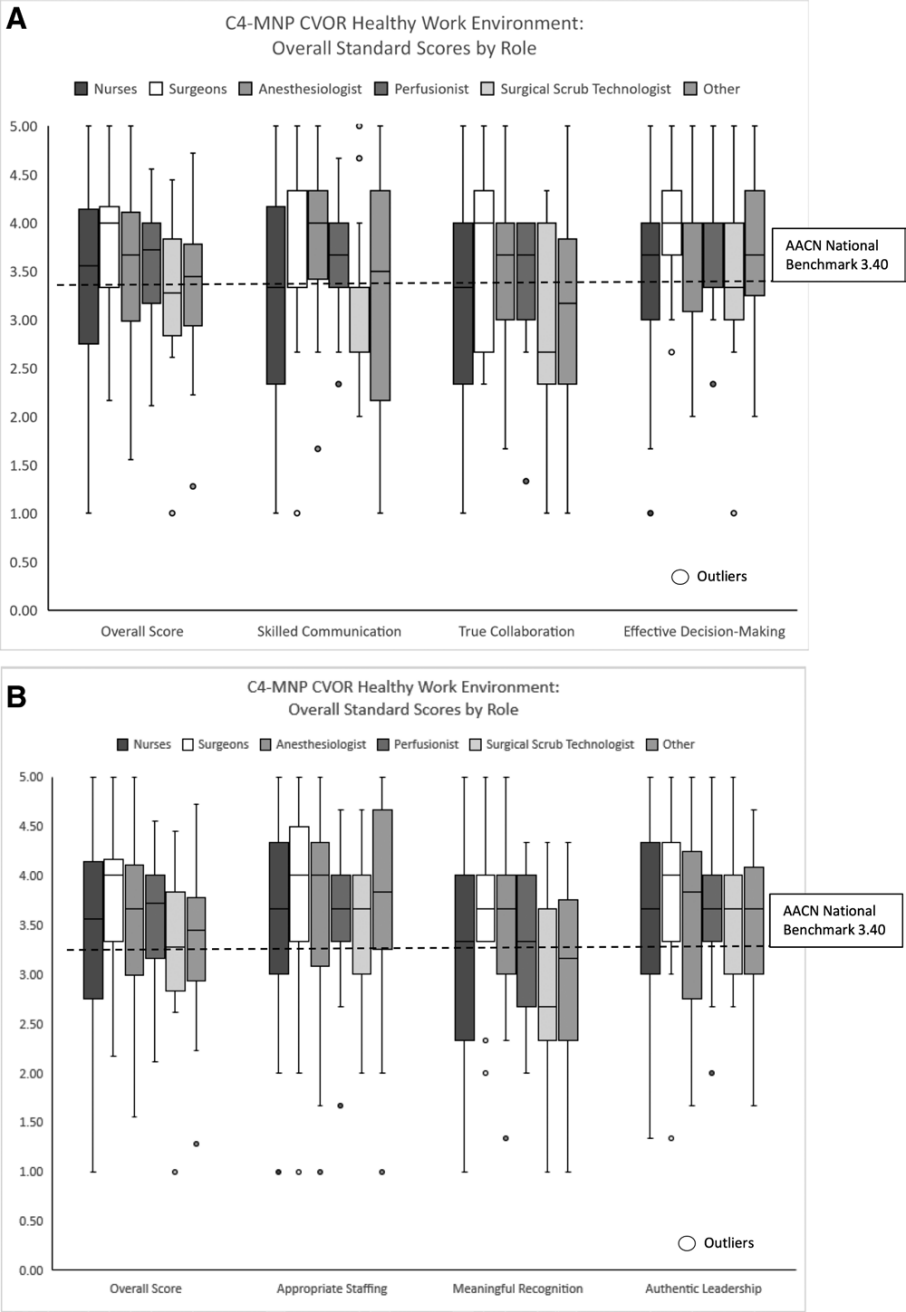
CVOR Healthy Work Environment Scores by Role vs AACN Benchmark. A and B, HWE survey: C4-MNP CVORs RN and surgeon scores with AACN national benchmark.

**Fig. 2.**
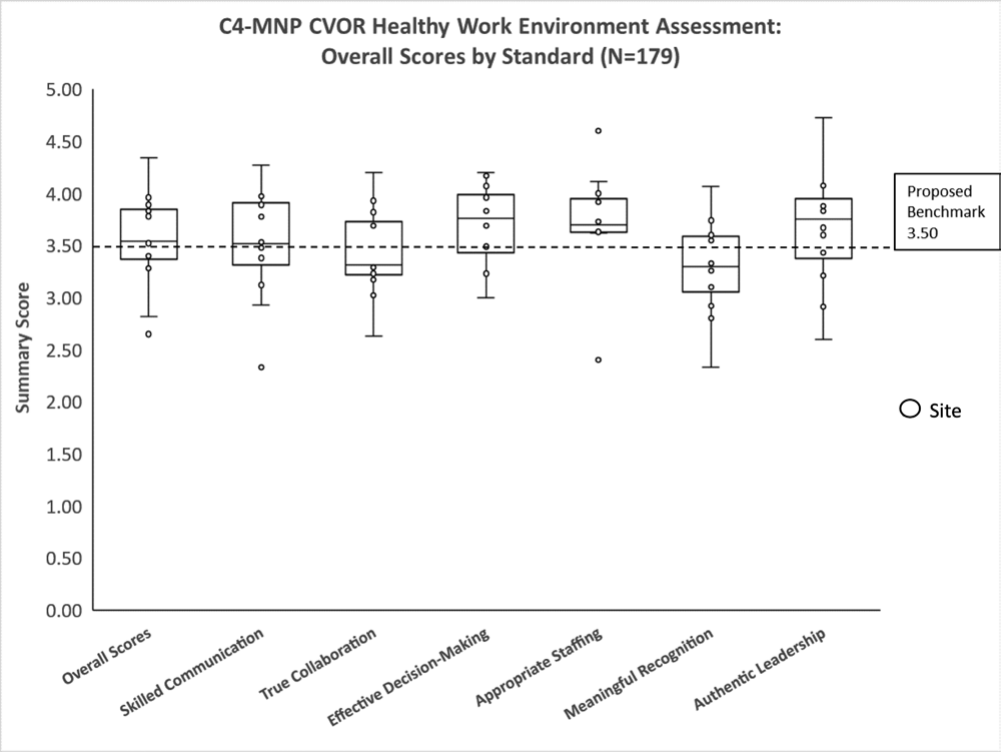
HWE survey: C4-MNP CVORs overall scores with proposed benchmark.

Findings from this project indicate that the pediatric CVOR work environment’s overall health was slightly better than the national AACN benchmark of 3.40; however, there was variation across sites (Table 2) with ranges from needs improvement to excellent. The average HWE score from 11 pediatric CVOR sites was 3.55, with a distribution of 2.65–4.34 (Table 2). Overall, the mean scores for each standard were within the “good” range. Participants reported the highest for effective decision-making and the lowest for meaningful recognition (Table 3). Analysis of HWE assessment by clinician role demonstrated that cardiovascular surgeons reported significantly higher overall HWE scores (M = 3.79, SD = 0.13) than nurses (M = 3.41, SD = 0.19; *P* = 0.02, two-tailed). A statistically significant difference in skilled communication *P* = 0.034, two-tailed. No significant difference was found between surgeons and RNs for the other five standards (Fig. 1A, B). Following the e-Delphi rounds, the consensus HWE pediatric CVOR benchmark was set at 3.50. Figure 2 illustrates overall pediatric HWE CVOR scores relative to the proposed benchmark.

**Table 2.** Distribution of HWE Scores by Site

	Overall Score	Skilled Communication	True Collaboration	Effective Decision-Making	Appropriate Staffing	Meaningful Recognition	Authentic Leadership
Overall	3.55	3.48	3.33	3.69	3.62	3.26	3.60
Site 1	4.34	4.27	4.20	4.20	4.60	4.07	4.73
Site 2	3.96	3.98	3.93	4.17	4.00	3.55	4.12
Site 3	3.89	3.78	3.70	3.96	4.11	3.74	4.07
Site 4	3.83	3.89	3.82	3.96	3.93	3.58	3.83
Site 5	3.78	3.97	3.69	4.07	3.67	3.60	3.67
Site 6	3.53	3.38	3.29	3.83	3.92	2.92	3.88
Site 7	3.52	3.50	3.26	3.69	3.65	3.13	3.91
Site 8	3.40	3.53	3.23	3.23	3.63	3.33	3.43
Site 9	3.28	3.12	3.02	3.49	3.73	3.10	3.21
Site 10	2.82	2.93	2.63	3.24	2.44	2.80	2.91
Site 11	2.65	2.33	3.17	3.00	2.40	2.33	2.60
HWE score interpretation							
1.00–2.99: need improvement		3.00–3.99: good			4.00–5.00: excellent		

**Table 3.** Pediatric CVOR HWE Assessment

Healthy Work Environment Assessment	M (Mean)	Range
Overall HWE	3.55	2.65–4.34
Skilled communication	3.48	2.33–4.27
True collaboration	3.33	2.63–4.20
Effective decision-making	3.69	3.00–4.20
Appropriate staffing	3.62	2.40–4.60
Meaningful recognition	3.26	2.33–4.07
Authentic leadership	3.60	2.60–4.73
HWE score interpretation		
1.00–2.99: need improvement	3.00–3.99: good	4.00–5.00: excellent

## DISCUSSION

This project provides a first-time use of the AACN HWEAT to describe the interprofessional health of work environments in pediatric CVORs in the United States. The aggregate pediatric CVOR scores indicated an overall “good” rating of 3.55. The lowest scores (mean 3.26) reported were for meaningful recognition, similar to the score reported by the AACN on this standard.^24^ Findings highlight that meaningful recognition may be an essential area to focus efforts to support and improve work environments across healthcare settings, including the pediatric CVOR. This assessment of pediatric CVORs also highlighted an area of strength when comparing scores with AACN HWE national assessment scores collected since 2010. Pediatric CVOR sites scored 0.31 higher than the national AACN scores reported on the Appropriate Staffing domain.^24^

A unique outcome of this project was that HWEAT data were collected from 11 pediatric CVOR sites, allowing for the first-time comparisons of work environments (Table 3). Although overall mean scores were “good” at 3.55, scores ranged from 2.65 to 4.34, indicating opportunities for targeted improvement. The standards with the lowest scores were skilled communication, true collaboration and meaningful recognition, which suggests an avenue to propose interventions to foster a healthier work environment. These data provide each site with invaluable information to evaluate their performance compared with peers. Comparing data between healthcare systems encourages performance improvement at the individual organizational level. Individual organizations would seek to improve their scores against other organizations and the collective scores of the study group.^25^ Subsequently, single organization improvement will cumulatively drive overall industry performance.^26^

Another important finding was the differences in perception of the HWE among surgeons and registered nurses. Surgeons reported higher overall HWE scores and higher scores for five essential standard scores, one of which was significantly different. These findings were similar to those reported by Connor et al.^16^ Although it is vital to understand interprofessional perceptions of the work environment, all leaders must also analyze data by clinical role.^5^ Based on these findings, it will be essential for cardiovascular program leaders to explore interprofessional differences before developing and implementing improvement initiatives.^5^ By using a comprehensive lens in data analysis and evaluation, leaders will be best positioned to implement and evaluate HWE improvement initiatives that target nursing concerns and have an interprofessional impact.

Data are vital for QI because they assess current systems, provide insight into what happens when changes are applied, and help us evaluate these changes’ success.^27^ Although many QI frameworks are available, an essential element among all frameworks is the need for metrics and benchmarks to drive QI initiatives.^28^ The C4-MNP collaborative forum aligns with this essential requirement by providing access to HWE data and encouraging utilization. The availability of HWE data for pediatric CVORs will enable program leaders and their interdisciplinary teams to meaningfully assess the health of their work environments and understand their scores in comparison to other pediatric CVORs across the United States. It empowers pediatric CVOR leaders with a forum to measure, compare, share and implement changes effectively, ultimately driving a culture of continuous improvement and ensuring the highest standards of care for pediatric cardiovascular patients nationwide.

**Fig. 3.**
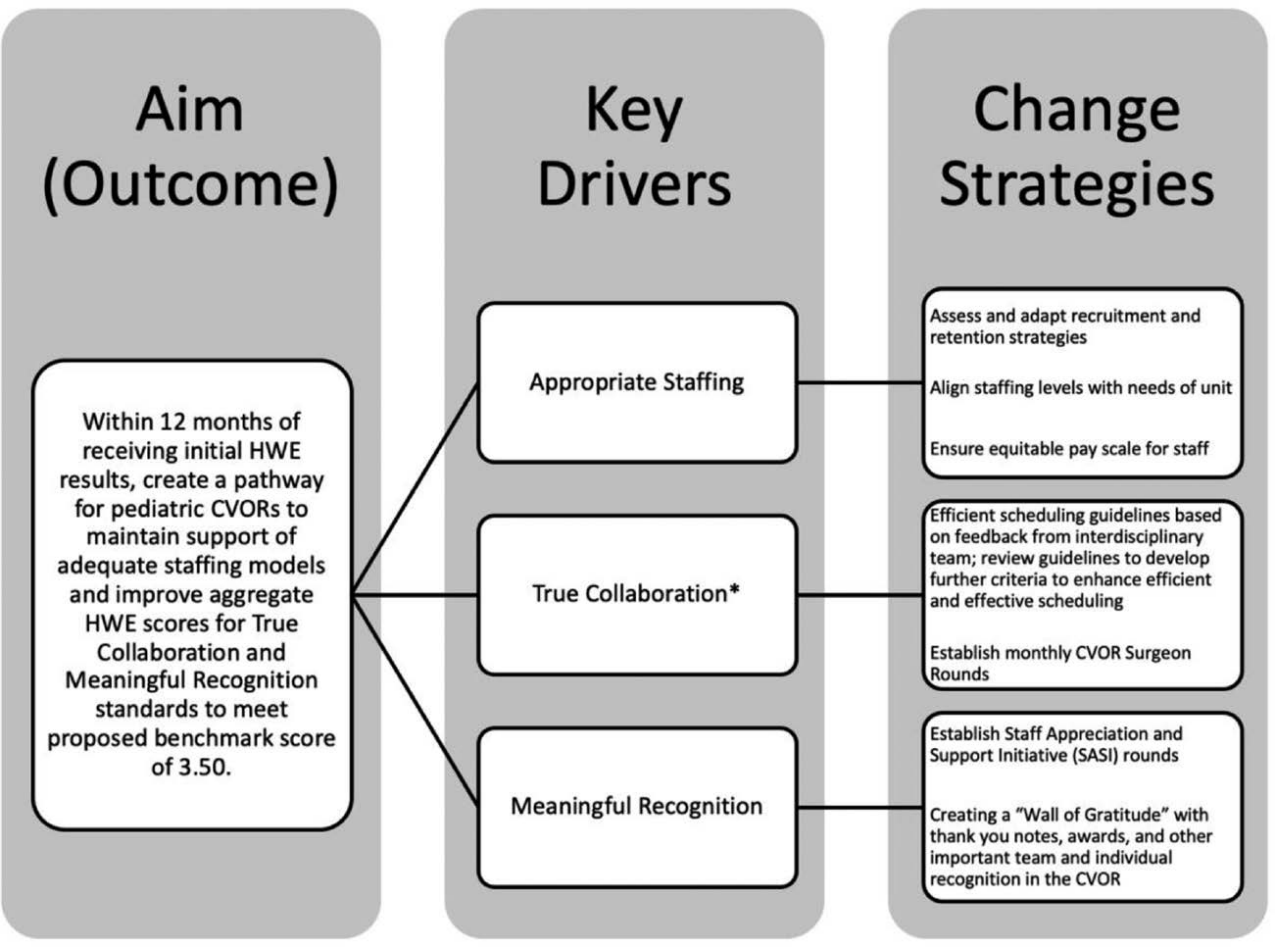
Key driver diagram. *AACN defines True Collaboration as a team that works together succeeds together. Collaboration among nurses and staff ensures more efficient, effective patient care and a more supportive environment where team members can develop in their practice.

The continued use of the HWEAT, targeted benchmarking in pediatric CVORs, and sharing of improvement initiatives within a dedicated collaborative forum will inform work environment improvement efforts in this challenging specialty. Figure 3 describes processes to utilize proposed change strategies to support adequate staffing models and improve HWE scores. Interventions must be adapted to fit the needs of individual sites.^29^ Assessing the health of their work environment will enable cardiovascular program leaders to evaluate targeted improvement strategies focused on any of the six AACN HWE standards, thus improving the health of their work environment in a meaningful and rapid manner.^29^ Involving frontline staff and interprofessional stakeholders is critical to the success of these initiatives. Multiple disciplines must collaborate in the perioperative area to provide high-quality, safe care.^30^ This work is meaningful due to the positive implications on staffing needs, healthcare organizations, and patient outcomes.^14,15^ Finally, the health of the work environment has important cost-saving implications due to the high monetary cost of recruiting and training new nurses to fill job vacancies.^6^

The limitations of survey design should be recognized. Participants may have been more compelled to participate if they were extremely satisfied or dissatisfied with their work environments, potentially overrepresenting only the far extremes of staff. Eleven freestanding pediatric CVORs across the United States participated, representing roughly 33% of the C4-MNP sites. Although this is a strong participation rate for a survey, participation from all sites would have resulted in more representative scoring of the health of the work environments across this specialty. We also recognize that the data were collected in 2017 and that the health of the work environment in these high-stress environments may have changed. Additionally, we recognize the difference in proportion by role, with surgeon respondents making up 11% of the sample. Continued use of the HWEAT in a larger group of CVORs, which is currently underway, will help support these consensus-based decisions and build empirical evidence in this area. As benchmarking continues through the growth of pediatric CVOR programs, results may look substantially different.

## CONCLUSIONS

This project provided a first-time use of the AACN HWEAT to describe the interprofessional work environments of pediatric CVORs. Project findings helped address a significant gap in the literature and provide a deeper understanding of the health of pediatric CVORs in the United States. Through collaboration and sharing among centers, best practices that support CVOR HWEs at high-performing centers can be applied at lower-performing centers. Creating and sustaining HWEs is essential for promoting quality and safety in patient outcomes and supporting nurse staffing models. Retaining perioperative nurses in specialty areas such as pediatric CVOR has never been more urgent than it is now, with the predicted shortages over the next few years.

## Supplementary Material


