# Supplementary material for: Measuring the Interprofessional Health of the Pediatric Cardiovascular Operating Room Work Environment
Source: Pediatr Qual Saf. 2024 Jun 11;9(3):e737. doi: 10.1097/pq9.0000000000000737 (PMC11167224; doi:10.1097/pq9.0000000000000737)

Supplemental Digital Content 1. AACN Standard for Establishing and Sustaining Healthy Work Environments

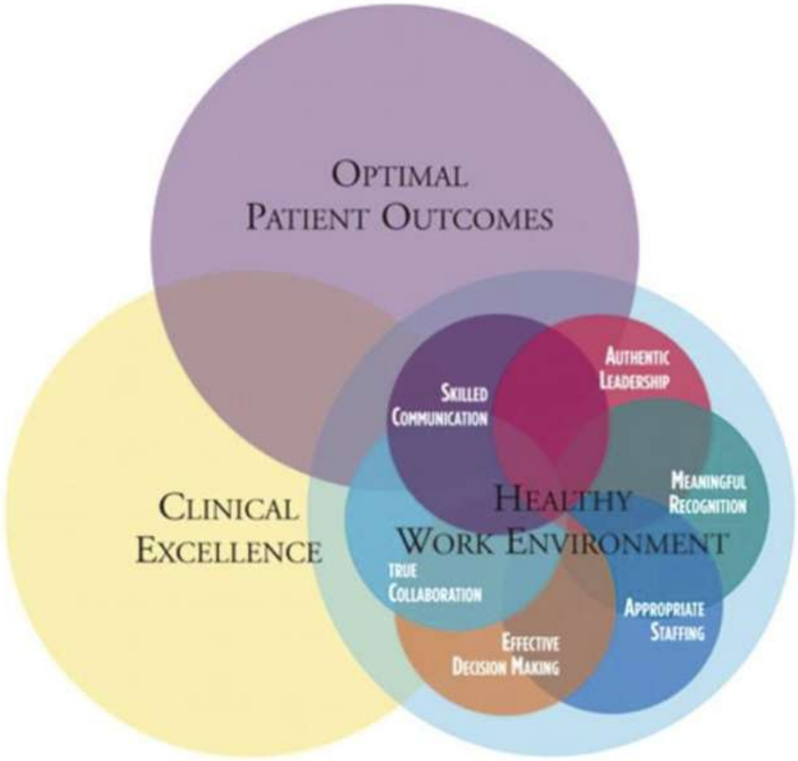

Supplement: Supplementary file 1 [file pqs-9-e737-s001.pdf]
