# Supplementary material for: Measuring the Interprofessional Health of the Pediatric Cardiovascular Operating Room Work Environment
Source: Pediatr Qual Saf. 2024 Jun 11;9(3):e737. doi: 10.1097/pq9.0000000000000737 (PMC11167224; doi:10.1097/pq9.0000000000000737)
Supplement: Supplementary file 2 [file pqs-9-e737-s002.pdf]

Supplemental Digital Content 2. AACN Healthy Work Environment Assessment

The AACN Healthy Work Environment Assessment measures the current health of your work environment against the American Association of Critical-Care Nurses Standards for Establishing and Sustaining Healthy Work Environments. The standards uniquely identify systemic behaviors that are often discounted, despite compelling evidence that they contribute to creating unsafe conditions and obstruct the ability of individuals and organizations to achieve excellence. The six standards are:

1. **Skilled Communication**: Nurses must be as proficient in communication skills as they are in clinical skills.
2. **True Collaboration**: Nurses must be relentless in pursuing and fostering true collaboration.
3. **Effective Decision Making**: Nurses must be valued and committed partners in making policy, directing, and evaluating clinical care and leading organizational operations.
4. **Appropriate Staffing**: Staffing must ensure the effective match between patient needs and nurse competencies.
5. **Meaningful Recognition**: Nurses must be recognized and must recognize others for the value each brings to the work of the organization.
6. **Authentic Leadership**: Nurse Leaders must fully embrace the imperative of a healthy work environment, authentically live it, and engage others in its achievement.

Administering the Assessment

The 18-question assessment should not take longer than 10 minutes to complete.

Participants are asked to indicate how much they agree or disagree with 18 statements related to the six AACN Standards for Establishing and Sustaining Healthy Work Environments on a scale of 1 to 5:

1	2	3	4	5
Strongly Disagree	Disagree	Neutral	Agree	Strongly Agree

	AACN Healthy Work Environment Assessment<br>**Please indicate how much you agree or disagree with the following statements**
1	Administrators, nurse managers, physicians, nurses and other staff maintain frequent communication to prevent each other from being surprised or caught off guard by decisions.<br>1 Strongly Disagree · 2 Disagree · 3 Neutral · 4 Agree · 5 Strongly Agree
2	Administrators, nurse managers, and physicians involve nurses and other staff to an appropriate degree when making important decisions.<br>1 Strongly Disagree · 2 Disagree · 3 Neutral · 4 Agree · 5 Strongly Agree
3	Administrators and nurse managers work with nurses and other staff to make sure there are enough staff to maintain patient safety.<br>1 Strongly Disagree · 2 Disagree · 3 Neutral · 4 Agree · 5 Strongly Agree
4	The formal reward and recognition systems work to make nurses and other staff feel valued.<br>1 Strongly Disagree · 2 Disagree · 3 Neutral · 4 Agree · 5 Strongly Agree
5	Most nurses and other staff here have a positive relationship with their nurse leaders (managers, directors, advanced practice nurses, etc.).<br>1 Strongly Disagree · 2 Disagree · 3 Neutral · 4 Agree · 5 Strongly Agree
6	Administrators, nurse managers, physicians, nurses, and other staff make sure their actions match their words— they "walk their talk."

	1 Strongly Disagree 2 Disagree 3 Neutral 4 Agree 5 Strongly Agree
7	Administrators, nurse managers, physicians, nurses, and other staff are consistent in their use of data-driven, logical decision-making processes to make sure their decisions are the highest quality.<br>1 Strongly Disagree 2 Disagree 3 Neutral 4 Agree 5 Strongly Agree
8	Administrators and nurse managers make sure there is the right mix of nurses and other staff to ensure optimal outcomes.<br>1 Strongly Disagree 2 Disagree 3 Neutral 4 Agree 5 Strongly Agree
9	Administrators, nurse managers, physicians, nurses, and other staff members speak up and let people know when they've done a good job.<br>1 Strongly Disagree 2 Disagree 3 Neutral 4 Agree 5 Strongly Agree
10	Nurses and other staff feel able to influence the policies, procedures, and bureaucracy around them.<br>1 Strongly Disagree 2 Disagree 3 Neutral 4 Agree 5 Strongly Agree
11	The right departments, professions, and groups are involved in important decisions.<br>1 Strongly Disagree 2 Disagree 3 Neutral 4 Agree 5 Strongly Agree
12	Support services are provided at a level that allows nurses and other staff to spend their time on the priorities and requirements of patient and family care.<br>1 Strongly Disagree 2 Disagree 3 Neutral 4 Agree 5 Strongly Agree
13	Nurse leaders (managers, directors, advanced practice nurses, etc.) demonstrate an understanding of the requirements and dynamics at the point of care, and use this knowledge to work for a healthy work environment.<br>1 Strongly Disagree 2 Disagree 3 Neutral 4 Agree 5 Strongly Agree
14	Administrators, nurse managers, physicians, nurses, and other staff have zero-tolerance for disrespect and abuse. If they see or hear someone being disrespectful, they hold them accountable regardless of the person's role or position.<br>1 Strongly Disagree 2 Disagree 3 Neutral 4 Agree 5 Strongly Agree
15	When administrators, nurse managers, and physicians speak with nurses and other staff, it’s not one-way communication or order giving. Instead, they seek input and use it to shape decisions.<br>1 Strongly Disagree 2 Disagree 3 Neutral 4 Agree 5 Strongly Agree
16	Administrators, nurse managers, physicians, nurses, and other staff are careful to consider the patient's and family's perspectives whenever they are making important decisions.<br>1 Strongly Disagree 2 Disagree 3 Neutral 4 Agree 5 Strongly Agree
17	There are motivating opportunities for personal growth, development, and advancement.<br>1 Strongly Disagree 2 Disagree 3 Neutral 4 Agree 5 Strongly Agree
18	Nurse leaders (managers, directors, advanced practice nurses, etc.) are given the access and authority required to play a role in making key decisions.<br>1 Strongly Disagree 2 Disagree 3 Neutral 4 Agree 5 Strongly Agree
